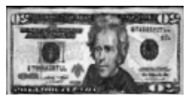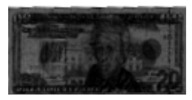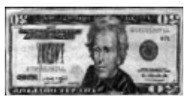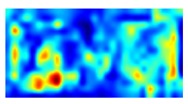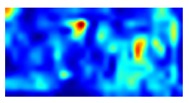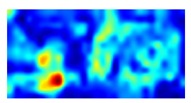# Joint Banknote Recognition and Counterfeit Detection Using Explainable Artificial Intelligence

**DOI:** 10.3390/s19163607

**Published:** 2019-08-19

**Authors:** Miseon Han, Jeongtae Kim

**Affiliations:** Department of Electronics and Electrical Engineering, Ewha Womans University, Seoul 03760, Korea

**Keywords:** banknote recognition, counterfeit banknote detection, explainable artificial intelligence, joint banknote recognition, counterfeit detection system

## Abstract

We investigated machine learning-based joint banknote recognition and counterfeit detection method. Unlike existing methods, since the proposed method simultaneously recognize banknote type and detect counterfeit detection, it is significantly faster than existing serial banknote recognition and counterfeit detection methods. Furthermore, we propose an explainable artificial intelligence method for visualizing regions that contributed to the recognition and detection. Using the visualization, it is possible to understand the behavior of the trained machine learning system. In experiments using the United State Dollar and the European Union Euro banknotes, the proposed method shows significant improvement in computation time from conventional serial method.

## 1. Introduction

Despite decreasing use of paper money and rapidly increasing electronic transactions, banknotes remain essential because they are easily carried, widely accepted, and reliable. For monetary transactions, it is critical that machines such as banknote counting and automatic teller machines accurately recognize banknote type and detect counterfeiting. For this reason, many studies have been conducted on banknote recognition and counterfeit detection [1,2,3,4,5].

Banknote recognition aims to identify banknote denomination and direction. Most banknote recognition methods follow the following steps [1]. First, banknote images are acquired using some sensors such as visible light and infrared sensors. Then, preprocessing crops proper image sections [1] and feature extraction is carried out to derive useful information from the cropped banknote images [1]. Finally, classification using the extracted features is performed to recognize banknote direction and classify banknotes into distinct denomination categories [1].

Conventional banknote recognition methods rely on digital image processing techniques, e.g., wavelet transforms, to extract features for classification [1]. However, designing high accuracy recognition methods is complicated because there can be many different kinds of damaged banknotes which should be recognized correctly [6]. In addition, recognition should be performed real time for most banknote recognition machines.

In contrast to digital image processing methods, machine learning methods, commonly employing convolutional neural networks (CNNs), can extract useful features without handcrafted feature extraction methods [7]. CNN based methods such as GoogleNet [8], DenseNet [9] and MobileNets [10] showed excellent performance in image classification task. Accordingly, various CNN based methods have been applied for banknote recognition and counterfeit detection [1,2,3,4,11,12,13]. Although these previous investigations were reported to perform well, computational requirements were demanding for real time implementation on embedded computers since network size were large [14].

Conventional counterfeit detection methods usually perform the detection after banknote recognition, using banknote information to extract useful features for counterfeit detection. For applying machine learning based methods, serial processing banknote recognition and counterfeit detection may require longer computational time than parallel processing [14]. Note that for most automatic teller machines, it is important to perform banknote recognition and counterfeit detection in real time without requiring large computational resource. In addition, serial banknote recognition and counterfeit detection may be error prone since recognition errors may lead to counterfeit detection failure.

To overcome the problems, we propose a novel joint banknote system to simultaneously perform banknote recognition and counterfeit detection. Because the proposed method shares convolutional layers for banknote recognition and counterfeit detection, we believe that the proposed method can be much faster than serial systems.

One of the most well known problems for machine learning classification methods is the difficulty to understand why the system generated such classification results [15]. If the machine learning system is not understandable, it may generate unexpected classification for testing data which were unseen during training. Understanding is very important to ensure confidence in the trained machine learning system. Although there have been many investigations on visualization such as classification activation map (CAM) and gradient weighted class activation map (Grad-CAM), none have been applied for machine learning based banknote recognition and counterfeit detection [16,17,18,19]. This paper is the first attempt to apply visualization methods for banknote recognition and counterfeit detection. Furthermore, we improve the traditional Grad-CAM method and propose pixel-wise Grad-CAM (pGrad-CAM) for clearer explanation for the resulting the proposed joint banknote recognition and counterfeit detection outcomes.

The remainder of this paper is organized as follows. Section 2 explains and analyzes related works in serial banknote recognition and counterfeit detection system and explainable artificial intelligence method. Section 3 summarizes contributions of this paper. Section 4 discusses two methods in detail: the proposed joint banknote recognition and counterfeit detection system and a new explainable artificial intelligence method. Section 5 experimentally demonstrates the usefulness of the proposed methods using the United State Dollar (USD) and the European Union Euro (EUR) banknotes. Section 6 summarizes and concludes the paper.

## 2. Related Works

### 2.1. Sequential Banknote Recognition and Counterfeit Detection System

One previous investigation studied a CNN based multinational banknote recognition considering size information for each banknote [5]. To reduce complexity of banknote recognition system, the method pre-classified banknote type by size, and then adopted separate CNN classifiers according to the size of the banknote [5]. Another machine learning method studied to recognize multinational banknote types using a CNN based model [3]. To handle the huge number of banknote classes, the method used pre-trained deep CNN models, AlexNet [7], GoogleNet [8], ResNet-18 and ResNet-50 [20]. Note that the pre-trained models were designed for image classification on the ImageNet database, which required an unnecessarily complicated structure for banknote recognition. Another study was conducted on banknote recognition using banknote images taken by a cellphone camera [21]. The method is based on the large frame Single Shot MultiBox Detector model [21] for banknote recognition and CNN for noise reduction [12]. Although the method showed higher performance than MobilNets [10] and faster R-CNN for banknote recognition, it suffered from real-time computation [12].

For counterfeit banknote detection, in addition to a visible light image, additional modality images such as infrared, ultraviolet, and magnetic images are often used because they provide useful information to help detect counterfeit notes [1]. Under the assumption that banknote type was correctly determined, features useful to detect counterfeit banknotes are extracted from manually predetermined regions of interest. Several methods have been studied for feature extraction, including bit plane slicing and canny edge detection [22] and luminance histogram and gray level co-occurrence matrix (GLCM) [1,23]. Using the features, counterfeit detection is performed by methods such as template matching [24] and key point matching [1,25]. Since handcrafted counterfeit detection methods require extensive efforts whenever new counterfeit types appear, machine learning based methods that can automatically learn new counterfeit types are highly desirable [4]. One previous investigation studied a CNN based counterfeit detection and proved performance using counterfeits made by general-purpose scanners [4]. Although the method achieved 100% detection accuracy [4], it included very few the Republic of Korea 10,000 Won banknotes and only considered counterfeit banknotes copied by general-purpose scanners. Another investigation studied detection of counterfeit Indian banknote using VGG16 based CNN [13]. Although the detection was successful, dataset was not sufficient to verify the performance of the method [13].

We summarize the pros and cons of conventional machine learning based sequential banknote recognition and counterfeit detection methods in comparison with joint method which we propose in this investigation. Conventional serial methods have two sequential neural networks for banknote recognition and counterfeit detection, respectively. It is relatively easy to train the networks because the loss function for each network has only single term. However, the sequential method usually requires longer computation time than the joint method due to its sequential nature [14]. The joint method which we propose has the advantage of fast computation thanks to simultaneous banknote recognition and counterfeit detection. Moreover, we design a compact CNN to accomplish faster joint banknote recognition and counterfeit detection. The fast computation of the joint method is achieved at the cost of more elaborated learning since training joint networks requires tuning of weight parameters between loss functions. One may come up with a joint method using well known CNN models for image classification task such as GoogleNet [8], DenseNet [9], and MobileNets [10]. To do that, one can modify the softmax layer of the models for joint banknote recognition and counterfeit detection and redesign loss function for training. Such modified models are very slow since the size of network is huge. Note that the CNN models are designed not for banknote recognition but for image classification task [8,9,10]. Table 1 summarizes pros and cons of sequential method, joint banknote recognition and counterfeit detection using models for image classification and proposed joint banknote recognition and counterfeit detection method.

### 2.2. Grad-CAM

Deep learning systems are generally difficult to interpret why the system generated such prediction [15]. It is desirable that the system is interpretable because inexplainable systems could generate unexpected predictions, and certainty is critical for banknote recognition and counterfeit detection. One of the most useful methods to interpret image based deep learning system is a visualization technique that highlights input image regions that contributed to the prediction. Among many visualization methods, Grad-CAM [16] is one of the most widely accepted methods. The Grad-CAM generates a localization map that highlights regions with large influence on the logit value from the last convolutional layer of CNN because feature maps derived from the layer have the most distinguishable features while retaining the spatial information.

The Grad-CAM method first computes the importance weight $\alpha_{k}^{c}$ as follows:

(1) $$\alpha_{k}^{c}=\frac{1}{MN}\sum_{i}\sum_{j}\frac{\partial y^{c}}{\partial A_{ij}^{k}},$$

where $y^{c}$ is the logit value for target class *c* and $A^{k}$ represents M×N size *k*-th feature map of the last convolutional layer. The importance weight $\alpha_{k}^{c}$ is computed by average pooling over the feature map $A^{k}$. Using the importance weights, Grad-CAM $L_{Grad-CAM}^{c}$ as follows:

(2) $$L_{\text{Grad-CAM}}^{c}=\mathrm{ReLU}\left(\sum_{k}\alpha_{k}^{c}A^{k}\right).$$

Note that the Grad-CAM is generated by computing weighted sum of feature maps where weight for each feature map is computed by the average of gradient values on the feature map. Grad-CAM considers only positive influences on the class of interest by applying ReLU operation after combining feature maps using the importance weights.

**Figure 1:**
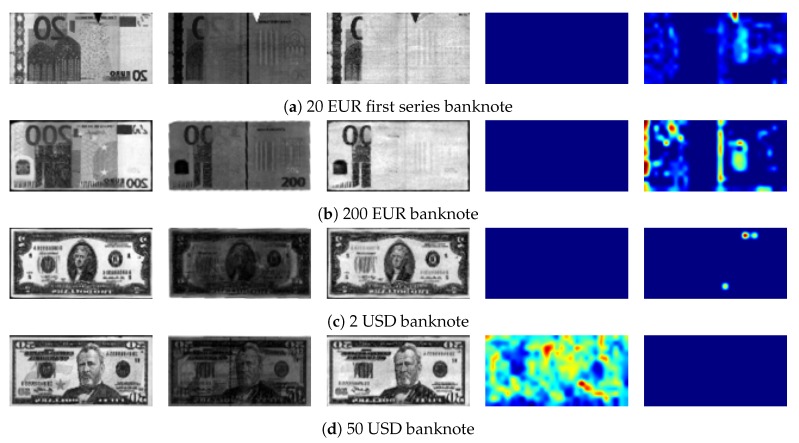
Grad-CAM results for some example banknotes. The most left column to the third column show visible, infrared transmission, and infrared reflection images of banknotes. The forth column and the most right column show Grad-CAM results for banknote recognition and counterfeit detection, reflectively.

Although Grad-CAM has been successfully applied for various applications [26,27,28], we think that the method should be improved because Grad-CAM generates empty activation maps for some cases. This happens when almost $\alpha_{k}^{c}$ values are negative values, because negative gradients values cancel positive gradient values during average-pooling. Figure 1 shows such example images which have predicted probabilities higher than 0.99 for both banknote recognition and counterfeit detection. Although the images can be clearly classified, as the high probabilities suggest, Grad-CAM activation maps were empty.

## 3. Contributions

The novelties of the proposed method are joint banknote recognition and counterfeit detection system and explainable artificial intelligence method for banknote recognition and counterfeit detection. To our knowledge, the proposed method is the first method which simultaneously classified banknote denomination, and counterfeit detection. Previous methods rely on denomination results to detect counterfeit banknote, which is slow. For the joint banknote recognition, and counterfeit detection, we use aligned visible, infrared reflection, and infrared transmission images as input data. We also proposed a novel loss function which combines three loss functions for banknote denomination, banknote direction, and counterfeit detection. Furthermore, our research is the first attempt to ensure the performance of banknote recognition method using explainable artificial intelligence. To do that, we propose a novel visualization method for banknote recognition and counterfeit detection system that overcomes shortcomings of an existing method. Note that we do not propose a novel CNN structure for banknote recognition system. The novelties of the proposed method are joint banknote recognition and counterfeit detection system and a visualization method to explain the system.

## 4. Methods

### 4.1. Joint Banknote Recognition and Counterfeit Detection System

**Figure 2:**
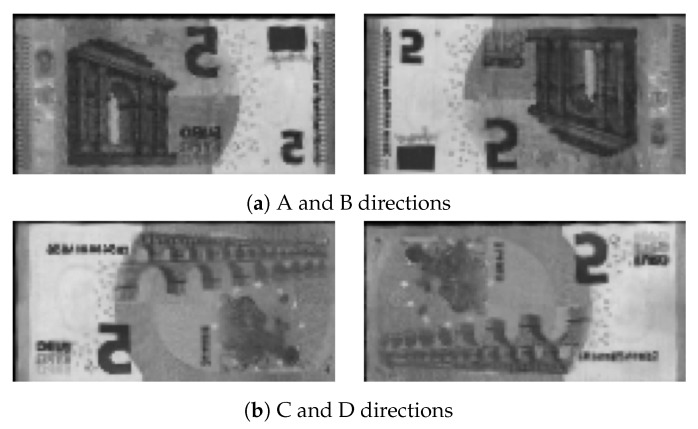
Possible banknote directions.

Conventional systems sequentially perform banknote recognition and counterfeit detection since counterfeit detection relies on the result of recognition [1]. Banknote recognition requires not only classifying the banknote denomination, but also its direction, because directional information is used for tasks such as banknote serial number recognition [1]. Figure 2 shows direction can be classified into four categories A, B, C, and D: front view, upside down (flipped), back to front (flopped), and flipped and flopped, respectively.

**Figure 3:**
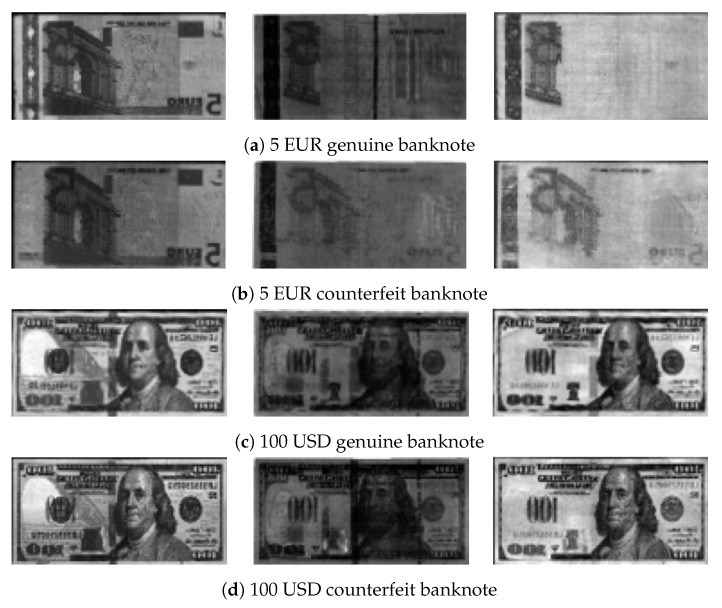
Different modality images. Leftmost column shows visible, center column shows infrared transmission, and rightmost column shows infrared reflection images.

Counterfeit detection detects forged banknotes based on features that only occur on genuine banknotes. Figure 3 shows examples of genuine features. Genuine EUR banknotes include an infrared transmission image with a center strip, which is not visible on the counterfeit banknote image while genuine USD banknotes have a center dashed strip on the infrared reflection image. To detect counterfeit features, some previous methods use digital image processing techniques. It is also possible to detect using machine learning based method. However, all previous methods use the information of banknote type to detect features for counterfeit detection.

**Figure 4:**
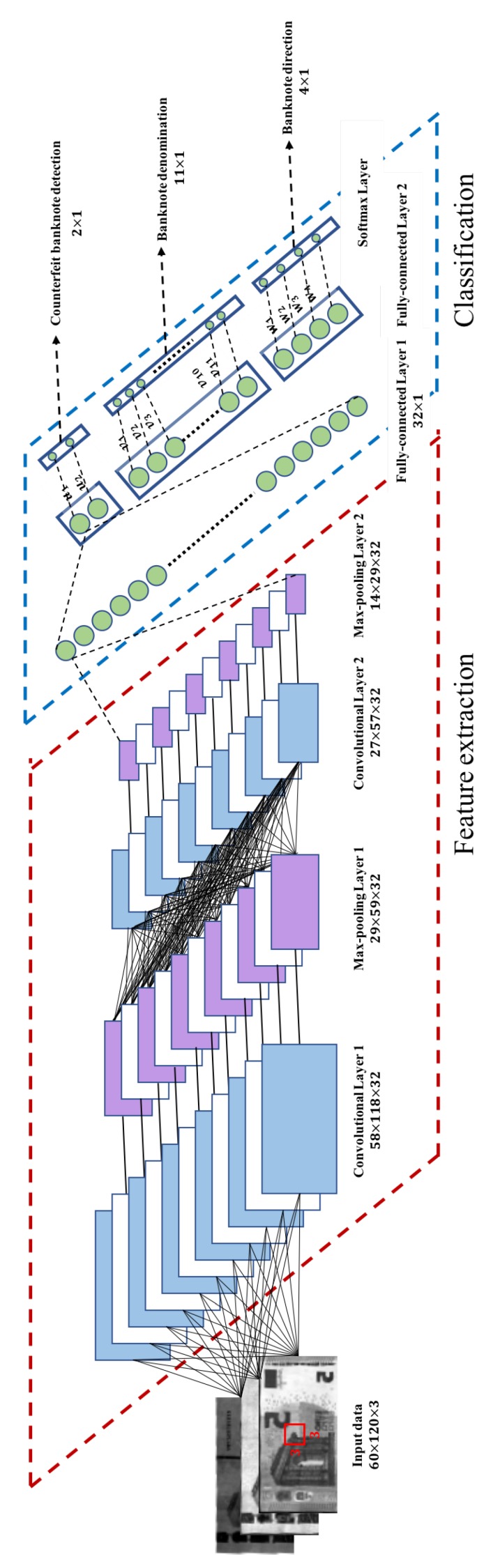
Joint banknote recognition and counterfeit detection system.

As pointed in the above, sequential banknote recognition and counterfeit detection can be slow since shallow networks are usually faster than deep networks [14]. To overcome the problem, we propose a novel joint banknote system that can simultaneously perform banknote recognition and counterfeit detection, as shown in Figure 4. The joint system consists of two convolutional layers, two max-pooling layers, and two fully connected layers followed by a softmax layer. We also used a batch normalization layer to avoid internal covariance shift [29], and the well known rectified linear unit (ReLU) nonlinear activation layer. Although there exist sophisticated nonlinear activation functions such as leaky ReLU [30], ReLU was sufficient to accomplish desired performance for the proposed network. Furthermore, ReLU is known to be more robust to noise than leaky ReLU [31,32].

We use the first convolutional layer with 32 feature maps and 3×3 convolution kernels to extract the features from three channel input data with a visible, an infrared reflection, and an infrared transmission images. After a batch normalization and a ReLU layer, convolved feature maps are subsampled by max-pooling with stride 2. Then, we use the subsampled feature maps as the input of the second convolutional layer with 32 feature maps and 3×3 convolution kernels. The output of the second convolutional layer is connected to first fully-connected layer with 32 nodes after a series of batch normalization, ReLU, and max-pooling layer with stride 2. The second fully connected layer generates logit values for banknote denomination, direction, and counterfeit detection. Then, the logit values are converted into probabilities using softmax layers as follows:

(3) $$p_{i}^{c}=\frac{e^{u_{i}}}{\sum_{i}e^{u_{i}}},\;i=0,1,$$

where $p_{i}^{c}$ denotes the probability of the prediction for counterfeit detection and

(4) $$p_{i}^{n}=\frac{e^{v_{i}}}{\sum_{i}e^{v_{i}}},\;i=0,\cdots ,N-1,$$

where $p_{i}^{n}$ denotes the probability of the prediction for banknote denomination and *N* is the number of banknote denominations for a country.

(5) $$p_{i}^{d}=\frac{e^{w_{i}}}{\sum_{i}e^{w_{i}}},\;i=0,\cdots ,3,$$

where $p_{i}^{d}$ is the probability of the prediction for banknote direction.

The proposed system was trained by minimizing a loss function combining $L_{det}$ for counterfeit banknote detection, $L_{den}$ for banknote denomination, and $L_{dir}$ for banknote direction. Mathematically, the total loss function $L_{tot}$ is defined as follows:

(6) $$L_{tot}=\lambda_{1}L_{det}+\lambda_{2}L_{den}+\lambda_{3}L_{dir},$$

where $\lambda_{1}$, $\lambda_{2}$, and $\lambda_{3}$ control weights for the three loss functions. In addition, $L_{det}$ is defined as follows:

(7) $$L_{det}=\sum_{i=0}^{1}q_{i}^{c}\log p_{i}^{c},$$

where $q_{i}^{c}$ is the true labeled probability of input banknote data, and $p_{i}^{c}$ is the corresponding prediction from the network. The denomination loss function is defined by

(8) $$L_{den}=\sum_{i=0}^{N-1}q_{i}^{n}\log p_{i}^{n},$$

where $q_{i}^{n}$ represents labeled probability of each denomination, $p_{i}^{n}$ denotes the corresponding prediction and *N* is the number of banknote denominations for a country. Finally, the direction loss function is defined by

(9) $$L_{dir}=\sum_{i=0}^{3}q_{i}^{d}\log p_{i}^{d},$$

where *i* is one of A, B, C, and D directions, $q_{i}^{d}$ is labeled probability for each direction and $p_{i}^{d}$ is the corresponding predicted probability.

The joint banknote recognition and counterfeit detection system predicts if the banknote is genuine or counterfeit. The joint system classifies banknote denomination where the number of classes depends on the number of different banknotes. The proposed system also predicts banknote direction. Note that system architecture can vary slightly for different national banknotes.

### 4.2. Explainable Artificial Intelligence

To resolve the empty activation map problem with Grad-CAM mentioned in Section 2.2, we propose a pixel-wise gradient weighted class activation map (pGrad-CAM) for visualization. The idea of pGrad-CAM is that if a feature map has both positive and negative gradients on a testing logit value, regions of positive gradients should be taken into consideration pixel-wisely even if the average of the gradient values is negative. Thus, we first compute weight map $W_{k}^{c}$ of the *k*-th feature for *c*-th class as follows:

(10) $$W_{k}^{c}=\mathrm{ReLU}\left(G_{\sigma }*\nabla_{A^{k}}y^{c}\right),$$

where $y^{c}$ is a logit for a target class *c*, $A^{k}$ means *k*-th feature map, * means convolution operator and Gaussian smoothing operator $G_{\sigma }$ is defined as follows:

(11) $$G_{\sigma }\left(i,j\right)=\frac{1}{2\pi \sigma^{2}}e^{-\frac{i^{2}+j^{2}}{2\sigma^{2}}}$$

where σ is a standard deviation of the distribution and *i* and *j* are pixel locations over the feature map. Using Gaussian smoothing operator, we would like to reduce the difference between intensities of adjacent pixels and extract more global features.

Using a linearly weighted combination between influences and feature maps, we compute pGrad-CAM as follows:

(12) $$L_{\text{pGrad-CAM}}^{c}=\sum_{k}\left(W_{k}^{c}⊙A^{k}\right),$$

where ⊙ is the pixel-wise product operation. Since $W_{k}^{c}$ never becomes all zero values, if positive gradient values exists, then pGrad-CAM never generate an empty map.

**Figure 5:**
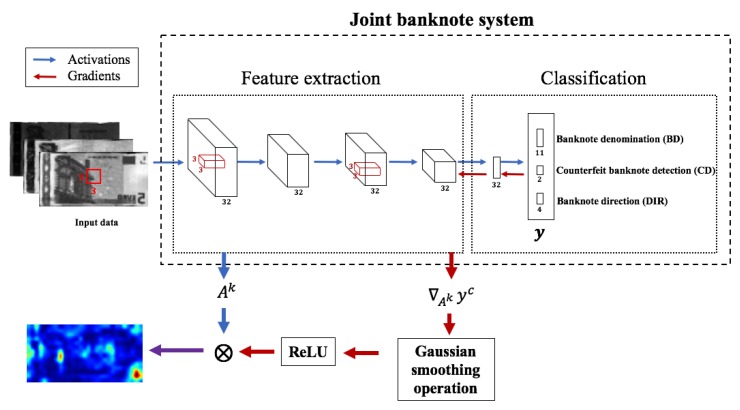
pGrad-Cam flow.

Figure 5 shows the block diagram to obtain pGrad-CAM. We first calculate the influence of the feature map extracted by the last convolutional layer on a logit through the gradient of the logit with respect to the feature map. We can identify the influence of each pixel on the logit $y^{c}$ due to pixel-wise operation. In addition, pGrad-CAM can highlight valid features using positive gradients without counterbalancing effects from negative influences on the logit. Using pGrad-CAM, we can understand how the network determines predictions and decides whether the model has high or low confidence.

## 5. Experimental Results

To evaluate the performance of the proposed method in comparison with sequential banknote recognition and counterfeit detection method, we conducted an experiment with EUR and USD banknotes. Genuine banknote images and counterfeit EUR and USD banknote images were acquired by Puloon Technology (Republic of Korea) using EagleEye10. The counterfeit EUR and USD images were acquired from the European Central Bank and the Federal Reserve Bank, respectively. We used 60×120 visible, infrared transmission, and an infrared reflection images for a total of 23,955 ×3 and 45,055 ×3 EUR and USD banknotes, respectively.

We implemented the proposed method as well as a sequential banknote recognition and counterfeit detection method. Further, for comparison purpose, we implemented a joint banknote recognition and counterfeit detection method using well known CNN for image classification. We selected GoogleNet which is composed of 22 layers [8] as the comparison method because the network showed high performance and fast inference time for image classification task [33]. We modified the final softmax layer of GoogleNet for the joint banknote recognition and counterfeit detection to compute the combined loss function defined in (6). We call this method by joint GoogleNet method.

We divided all banknote images into training, validation, and test datasets at 9:0.5:0.5 ratio, respectively. For training data, we flipped the counterfeit banknote images horizontally, vertically, and both horizontally and vertically for data augmentation. As the result, the augmented counterfeit dataset is four times larger than original counterfeit dataset. Table 2 summarizes dataset sizes for each banknote type. The training dataset of the EUR banknotes comprised 18,321 genuine and 3476 counterfeit banknotes (including augmentation) while the training dataset of the USD banknotes comprised 33,583 genuine and 7520 counterfeit banknotes (including augmentation).

**Figure 6:**
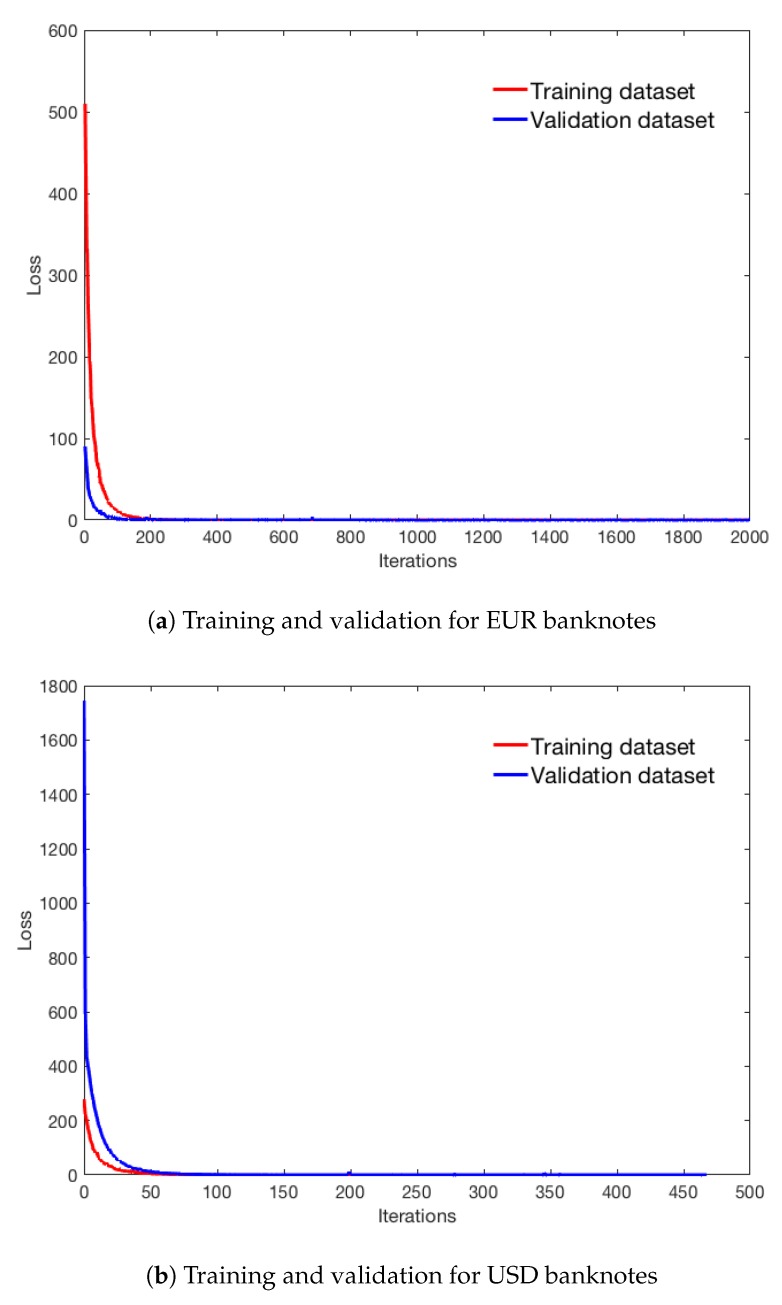
Average batch losses of convergence graphs.

We implemented the proposed and the sequential methods using Tensorflow and tested the trained model on an embedded NVIDIA Jet AGX system (NVIDIA, USA). For training network, we determined the weights of the loss function manually as $\lambda_{1}=2$, $\lambda_{2}=1$, and $\lambda_{3}=1$. To avoid boundary artifacts, we use convolution with valid option instead of convolution with same or full option [34]. In addition, to avoid overfitting, we applied 50% dropout between the last convolutional layer and the first fully connected layer. We applied dropout only between the last convolutional layer and the first fully connected layer because the second fully connected layer does not have many connections. Although one may apply dropout in convolutional layer too, it has been known that performance enhancing of dropout in convolution layer is minimal [35]. Note that the fully connected dropout is known as the best performer on a small architecture [36]. We trained the joint banknote recognition and counterfeit detection system using ADAM optimizer with 0.0001 adaptive learning rate, 0.9 decay factor, and 512 batch size for EUR while using ADAM optimizer with 0.0001 fixed learning rate and 512 batch size for USD [37]. We terminated training when training and validation losses were below the pre-determined threshold (0.005). Figure 6 shows the changes of average batch losses for training and validation datasets during training. The final average training and validation losses were $6.97\times 10^{-5}$ and $1.87\times 10^{-4}$ for EUR banknote network while $4.18\times 10^{-5}$ and $7.89\times 10^{-4}$ for USD banknote network.

Table 3 shows banknote recognition and counterfeit detection accuracies which are defined as the ratio of the number of correctly recognized and correctly classified into genuine or counterfeit to the total number of tested banknote images. For EUR and USD banknotes, all methods achieved 100% accuracies, which imply machine learning based method can perform very well for banknote recognition and counterfeit detection. This result is not surprising since machines which deal with currency must show extremely high accuracy. Note that we have tested real banknote images for this experiments. Needless to say, if the methods were tested using damaged banknotes, the proposed method as well as other methods may not show 100% accuracy. Investigation of performance for damaged banknote images is deferred to future study.

Although all methods showed the same accuracy, the proposed method was markedly faster than the other methods. Table 4 shows mean and variance of the computational time for 1000 times executions for each method. We used the same network as in the proposed method twice for the sequential banknote recognition and counterfeit detection method. The preprocessing time is computation time for image resizing and normalization. The total computational time of the sequential method was about 11.69 ms. Except for preprocessing, the sequential method required approximately 4.18 ms and 3.94 ms for banknote recognition and counterfeit detection, respectively. The joint GoogleNet required similar preprocessing. However, computation time for joint banknote recognition and counterfeit detection was about 947.12 ms, which is impractical to be used for banknote recognition. This is due to the joint GoogleNet has lots of layers and weight parameters, which may be necessary for image classification but not for banknote recognition. On the contrary, the total computational time of the proposed method is only about 8.09 ms. The proposed method took about 4.36 ms for the joint banknote recognition and counterfeit detection. One may think that performance improvement of 3.6 ms (difference between means of the sequential method and the proposed method) may not be important. However, the performance improvement is more than 30%, which can be critical for realtime implementation on an embedded machine.

Even though the proposed system achieved 100% accuracy for both banknote classification and counterfeit detection and improved speed compared with other methods, we believe it is essential for the system to be well explained to ensure confidence that the system will not generate unexpected outcomes for unseen data. To do that, we generated activation maps and identified regions with large effects on the prediction. Table 5 shows the activation maps of the joint system using Grad-CAM and pGrad-CAM.

In Table 5, from the most left column to the third column show visible, infrared transmission and infrared reflection images of banknotes. The fourth and fifth columns show Grad-CAM and pGrad-CAM results for banknote recognition while the sixth column and the most right column show Grad-CAM and pGrad-CAM results for counterfeit detection. In the Grad-CAM and the pGrad-CAM results, more reddish regions correspond to higher influence on the class whereas more bluish regions correspond to lower influence on the class.

Although Grad-CAM generated explainable results for most cases, the method failed for some EUR and USD banknotes as shown in Table 5. The probabilities of all banknotes in Table 5 have higher than 0.99 for both banknote recognition and counterfeit detection. However, Grad-CAM did not show anything for some cases (i.e., all zero values) such as 20 EURs, 200 EUR, 1 USD, and 2 USD for banknote recognition and 50 USD and 100 USD for counterfeit detection. In 500 EUR banknotes, although Grad-CAM results for banknote recognition have some colored pixels, the effects seem to be too restricted to understand the behavior of the network. Similar phenomena occur for 1 USD and 2 USD banknotes for counterfeit detection.

Unlike Grad-CAM, pGrad-CAM for 20 EUR first series and 20 EUR second series highlights the upper digits as very important clues to classify banknote denomination, which we believe reasonable. For 200 EUR banknote, pGrad-CAM highlights regions on the upper digits and the center of door in a visible image as important features for banknote recognition. For 500 EUR banknote recognition, pGrad-CAM highlights regions that contain important features such as regions near the left rectangular box. For 1 USD and 2 USD banknotes, pGrad-CAM highlights the center portrait and features near sides of the banknote. For counterfeit detection, Grad-CAM highlights similar regions highlighted in pGrad-CAM for EUR banknotes. However, Grad-CAM appears to emphasize irrelevant regions for USD banknotes such as near the margin.

Table 6 shows averaged Grad-CAM and pGrad-CAM results for banknote denomination, direction, and counterfeit detection. For EUR banknote denomination, it seems that averaged Grad-CAM highlights less important regions such as the upper right rectangular box for 20 EUR first series banknote and the bottom right rectangular box for 100 EUR banknote than pGrad-CAM. The pGrad-CAM stresses important features such as the upper digits for 20 EUR first series banknote and the door in the lower left region in 100 EUR banknote. For USD banknote denomination and EUR counterfeit detection, both Grad-CAM and pGrad-CAM highlight similar regions. For 10 USD banknote, Grad-CAM did not indicate lower left regions that have counterfeit features. In contrast, pGrad-CAM seems to highlight regions that contain important features for banknote recognition and counterfeit detection. We think this is because pGrad-CAM is based on the pixelwise effects of feature maps on prediction, which may provide more accurate information about regions that effect prediction.

## 6. Conclusions

This paper proposed a simultaneous banknote recognition and counterfeit detection system, with a related explainable artificial intelligence method. Experiments using USD and EUR banknotes confirmed the proposed method achieved significantly faster computation than the conventional sequential method while retaining 100% banknote recognition and counterfeit detection accuracy. The proposed pGrad-CAM structure also explained proposed network system behavior better than conventional Grad-CAM. Therefore, we believe that the proposed method will be useful for practical banknote recognition and counterfeit detection.

## Figures and Tables

**Table 1:** A summary comparison for pros and cons.

Methods	Pros	Cons
Sequential method	- Relatively easy to train	- Relatively long inference time
Joint method using CNN for image classification	-	- Extremely slow - Relatively difficult to train
Proposed method	- Fast inference time	- Relatively difficult to train

**Table 2:** Banknote datasets.

Nation	Denomination		The Number of Dataset					
			Train		Validation		Test	
	Series	Type	Genuine	Counterfeit	Genuine	Counterfeit	Genuine	Counterfeit
EUR	First	5 EUR	3266	8	182	1	182	1
		10 EUR	744	76	42	2	42	2
		20 EUR	634	140	36	3	36	3
		50 EUR	864	536	49	8	49	8
		100 EUR	3110	216	173	3	173	3
		200 EUR	824	1364	46	20	46	20
		500 EUR	967	644	54	9	54	9
	Second	5 EUR	1535	112	86	2	86	2
		10 EUR	2304	40	129	1	129	1
		20 EUR	1371	268	77	4	77	4
		50 EUR	2702	72	151	1	151	1
	Total		18,321	3476	1025	54	1025	54
USD	1 USD		1735	0	97	0	97	0
	2 USD		3780	0	210	0	210	0
	5 USD		2574	0	143	0	143	0
	10 USD		5298	1664	295	24	295	24
	20 USD		8369	5436	466	76	466	76
	50 USD		8263	340	460	5	460	5
	100 USD		3564	80	198	2	198	2
	Total		33,583	7520	1869	107	1869	107

**Table 3:** Accuracy of banknote system.

Nation	Dataset	Number of Banknotes (Counterfeit)	Number of Well-Classified (Counterfeit)	Accuracy (%)		
				Sequential Method	Joint GoogleNet	Proposed Method
EUR	Train and validation	22,876 (3530)	22,876 (3530)	22,876/22,876 (100)	22,876/22,876 (100)	22,876/22,876 (100)
	Test	1079 (54)	1079 (54)	1079/1079 (100)	1079/1079 (100)	1079/1079 (100)
USD	Train and validation	43,079 (7627)	43,079 (7627)	43,079/43,079 (100)	43,079/43,079 (100)	43,079/43,079 (100)
	Test	1976 (107)	1976 (107)	1976/1976 (100)	1976/1976 (100)	1976/1976 (100)

**Table 4:** Mean and variance of inference time.

Model	Processing Time on Average (variance)			
	Preprocessing	Banknote Recognition	Counterfeit Detection	Total
Sequential	3.57 ms (0.46)	4.18 ms (0.86)	3.94 ms (0.69)	11.69 ms (3.02)
Joint GoogleNet	3.53 ms (0.12)	947.12 ms (2817.44)		950.65 ms (2804.65)
Proposed	3.73 ms (0.49)	4.36 ms (0.72)		8.09 ms (1.18)

**Table 5:** Grad-CAM and pGrad-CAM results.

Banknote	Input Images			Banknote Recognition		Counterfeit Detection	
	Visible	Infrared Transmission	Infrared Reflection	Grad-CAM	pGrad-CAM	Grad-CAM	pGrad-CAM
20 EUR first series	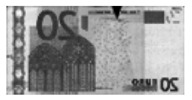	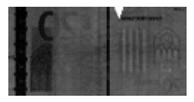	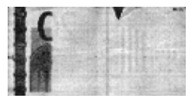	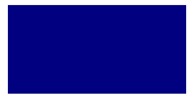	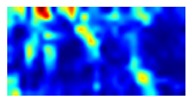	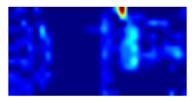	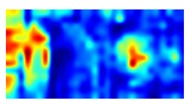
20 EUR second series	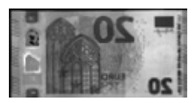	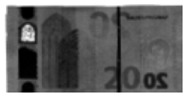	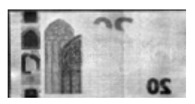	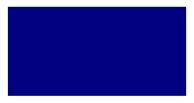	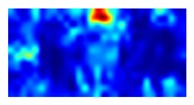	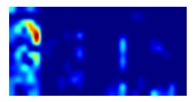	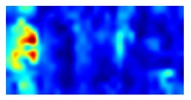
200 EUR	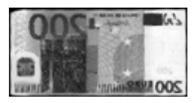	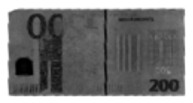	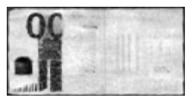	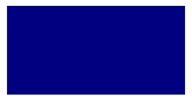	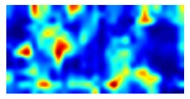	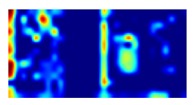	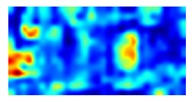
500 EUR	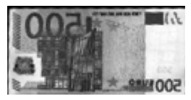	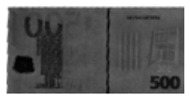	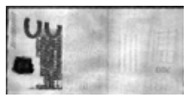	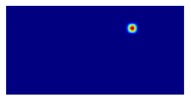	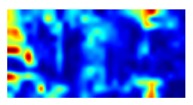	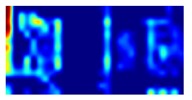	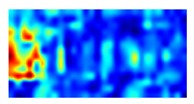
1 USD	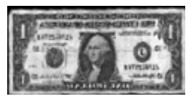	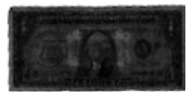	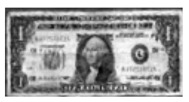	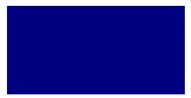	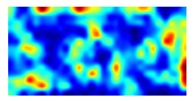	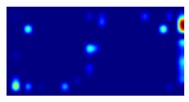	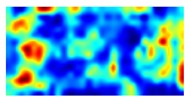
2 USD	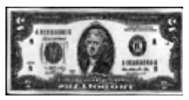	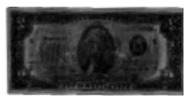	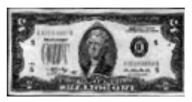	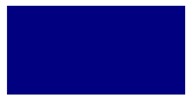	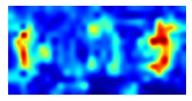	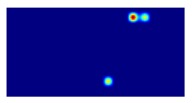	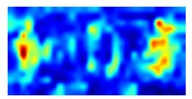
50 USD	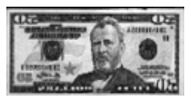	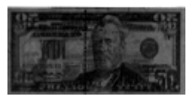	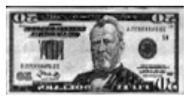	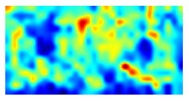	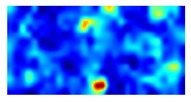	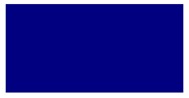	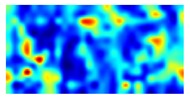
100 USD	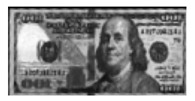	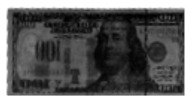	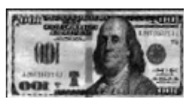	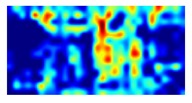	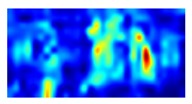	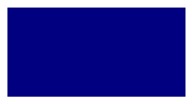	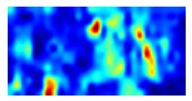

**Table 6:** Average Grad-CAM and pGrad-CAM results.

Banknote	Method	Input Example Images			Explainable Artificial Intelligence		
		Visible	Infrared Transmission	Infrared Reflection	Banknote Denomination	Banknote Direction	Counterfeit Detection
20 EUR first series	Grad-CAM	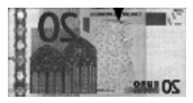	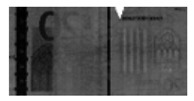	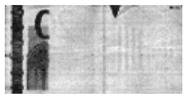	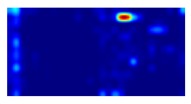	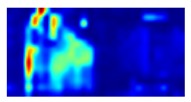	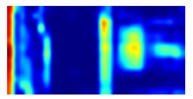
	pGrad-CAM	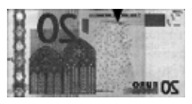	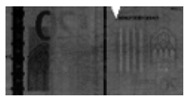	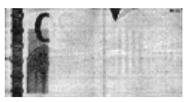	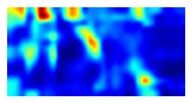	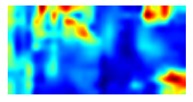	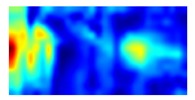
100 EUR	Grad-CAM	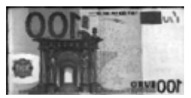	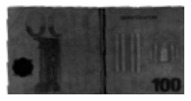	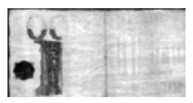	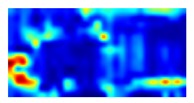	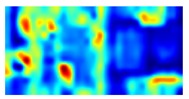	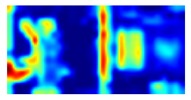
	pGrad-CAM	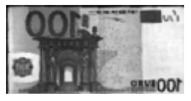	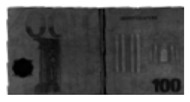	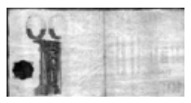	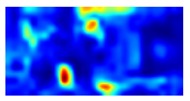	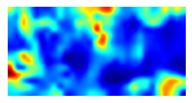	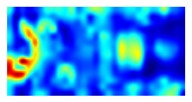
10 USD	Grad-CAM	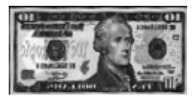	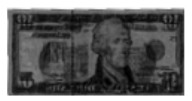	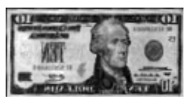	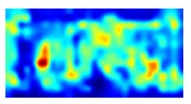	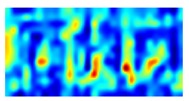	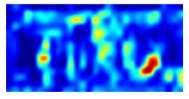
	pGrad-CAM	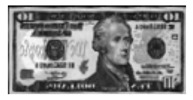	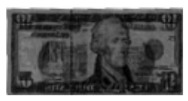	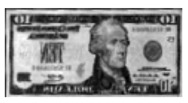	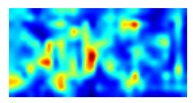	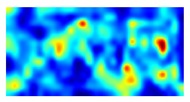	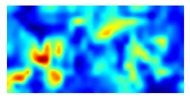
20 USD	Grad-CAM	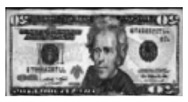	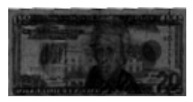	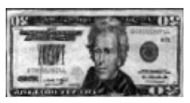	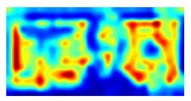	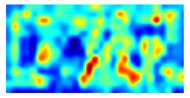	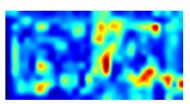
	pGrad-CAM